# Nomogram to predict ventilator-associated pneumonia in large vessel occlusion stroke after endovascular treatment: a retrospective study

**DOI:** 10.3389/fneur.2024.1351458

**Published:** 2024-05-13

**Authors:** Huishan Zhu, Wenfei Liang, Jingling Zhu, Xiaohua He, Pengjuan Zou, Kangqiang Yang, Guoshun Li, Bin Liao, Huiquan Deng, Zichong Liang, Jiasheng Zhao, Zhan Zhao, Jingyi Chen, Qiuxing He, Weimin Ning

**Affiliations:** ^1^Department of Neurology, Dongguan Hospital of Guangzhou University of Chinese Medicine, Dongguan, China; ^2^School of Traditional Chinese Medicine, Southern Medical University, Guangzhou, Guangdong, China; ^3^South China Research Center for Acupuncture and Moxibustion, Medical College of Acu-Moxi and Rehabilitation, Guangzhou University of Chinese Medicine, Guangzhou, China

**Keywords:** nomogram, ventilator-associated pneumonia, acute ischemic stroke with large vessel occlusion, endovascular treatment, mechanical ventilation

## Abstract

**Background:**

Ventilator-Associated Pneumonia (VAP) severely impacts stroke patients’ prognosis after endovascular treatment. Hence, this study created a nomogram to predict the occurrence of VAP after endovascular treatment.

**Methods:**

The individuals with acute ischemic stroke and large vessel occlusion (AIS-LVO) who received mechanical ventilation and endovascular therapy between July 2020 and August 2023 were included in this retrospective study. The predictive model and nomogram were generated by performing feature selection optimization using the LASSO regression model and multifactor logistic regression analysis and assessed the evaluation, verification and clinical application.

**Results:**

A total of 184 individuals (average age 61.85 ± 13.25 years, 73.37% male) were enrolled, and the rate of VAP occurrence was found to be 57.07%. Factors such as the Glasgow Coma Scale (GCS) score, duration of stay in the Intensive Care Unit (ICU), dysphagia, Fazekas scale 2 and admission diastolic blood pressure were found to be associated with the occurrence of VAP in the nomogram that demonstrating a strong discriminatory power with AUC of 0.862 (95% CI, 0.810–0.914), and a favorable clinical net benefit.

**Conclusion:**

This nomogram, comprising GCS score, ICU duration, dysphagia, Fazekas scale 2 and admission diastolic blood pressure, can aid clinicians in predicting the identification of high-risk patients for VAP following endovascular treatment in large vessel occlusion stroke.

## 1 Introduction

Endovascular treatment (EVT) is highly recommended for cases of acute ischemic stroke with large vessel occlusion (AIS-LVO) due to the urgency of the patient’s situation (1). As an anesthesia technique frequently used in EVT protocols, General Anesthesia (GA) with intubation provides the benefits of safeguarding the airway and complete patient immobilization, leading to enhanced angiographic image quality and ensuring surgical safety (2, 3). Nonetheless, mechanical ventilation (MV) often leads to the development of ventilator-associated pneumonia. Ventilator-associated pneumonia (VAP) is a type of pneumonia that occurs in individuals who have been intubated or undergone tracheostomy and have been on mechanical ventilation for either 48 h or within 48 h after extubation (4). Stroke patients with delayed extubation (>24 h) after mechanical thrombectomy showed a higher incidence of pneumonia than those who were extubated within 24 h (5). The most critical period for developing VAP is within the initial 5 days of mechanical ventilation (6). According to research conducted in China, the prevalence of VAP can reach up to 48.4% (7), accompanied by a maximum fatality rate of 43.2% (8, 9). The condition of AIS-LVO patients is critical and there are additional risk factors such as impaired consciousness, dysphagia and stroke-induced immunosuppression (SIIS) that are of particular relevance to a high incidence of pneumonia (10). Additionally, patients with mechanical ventilation after EVT would be transferred to the ICU or NICU wards for monitoring, which increases the exposure of patients to pathogenic bacteria, making them more susceptible compared to non-LVO patients. On the contrary, the occurrence of pneumonia can also lead to difficulties in extubation, prolonging ICU hospitalization, and increasing hospitalization costs (3). Furthermore, the presence of VAP can significantly impact the restoration of neurological function in individuals with stroke. Additionally, the inflammatory response can exacerbate the post-stroke brain injury, potentially resulting in severe complications like sepsis, multiorgan dysfunction, infectious shock, gastrointestinal hemorrhage, and even fatality (11). Therefore, pneumonia acts as a separate influential element that has an adverse impact on the prognosis of individuals with stroke, presenting a considerable obstacle to clinicians.

Early identification and intervention of VAP is of great significance for subsequent treatment and improvement of patient prognosis. In clinical practice, clinicians can use the prediction models of VAP in early identification, thus addressing this obstacle. Currently, there are various prediction models available for VAP prediction. Wu et al. proposed a model for predicting ventilator-associated pneumonia in individuals suffering from acute respiratory distress syndrome (12), while the model of Liao et al. predicts VAP in hospitalized patients with hypertensive intracerebral hemorrhage (13). However, there are currently few studies on the predictive model of VAP after EVT for stroke. As a result, the objective of this research was to develop a precise and personalized prediction nomogram to facilitate clinicians to identify the high-risk individuals for VAP, so that early intervention can be implemented, ultimately enhancing patient prognosis.

## 2 Methods

### 2.1 Study design and patients selection

The AIS-LVO patients in the Neurology Department of Dongguan Hospital of Traditional Chinese Medicine admitted from July 2020 to August 2023 who accepted mechanical ventilation and EVT were included as research subjects. All data of patients would be retrospectively collected for the construction of a predictive model. Participants for the study were selected according to the following criteria: (1) Age between 18 and 89 years, (2) presence of large vessel occlusion or severe stenosis indicated by digital subtraction angiography (DSA) onset-to-arrival time less than 24 h, and (3) recipient of endovascular treatment and general anesthesia with intubation. The exclusion criteria include: (1) anesthesia recipient of conscious sedation or local anesthesia, (2) preoperative pneumonia, (3) incomplete medical records, and (4) refusing follow-up or disconnection.

The research adheres to the Helsinki Declaration (updated in 2013) and conforms to the TRIPOD statement (Transparent Reporting of a Multivariable Prediction Model for Individual Prognosis or Diagnosis) (14). The research has been approved by the Ethics Committee of Dongguan Hospital, Guangzhou University of Chinese Medicine (PJ [2023] NO.63). Informed consent is not required since the study does not directly affect patients, and all private health information has been anonymized to safeguard patient confidentiality. The corresponding author can provide the supporting data for this study upon request. This study was not influenced by any commercial entities during its design, implementation, or reporting.

### 2.2 Primary and secondary clinical outcomes

The objective of this research was to create a nomogram capable of investigating the occurrence of VAP after EVT in stroke patients. To diagnose ventilator-associated pneumonia (VAP), certain criteria must be met (4), which involve the existence of new or deteriorating irregularities on chest X-ray or CT scan, in addition to a minimum of two of the subsequent clinical indications: (1) elevated body temperature exceeding 38°C, (2) respiratory secretions that are purulent, (3) peripheral blood leukocyte count that exceeds 10 × 10^9^/L or falls below 4 × 10^9^/L, and (4) presence of cough, dyspnea, or tachypnea (respiratory rate surpassing 25 breaths per minute). The study excluded patients with symptoms similar to those of other conditions, including tuberculosis, lung tumors, non-infectious lung diseases, pulmonary edema, pulmonary embolism, or lung collapse. The secondary focus is on the 90 days prognosis by assessing the modified Rankin Scale (mRS) score. An mRS score ranging from 0 to 2 suggests a favorable outcome, whereas a score ranging from 3 to 6 suggests an unfavorable outcome. All clinical data were evaluated by a trained neurologist in a blinded manner.

### 2.3 Predictor factor selection

Firstly, the authors conducted a comprehensive search of the existing literature to identify the relevant risk factors for VAP. After considering these risk factors and examining the clinical and laboratory data stored in this electronic health record system, a total of 36 potential variables were identified for inclusion in the modeling process. There is the demographic information (gender, age), medical history like hypertension, diabetes, coronary artery disease (CAD), atrial fibrillation, chronic obstructive pulmonary disease (COPD), previous stroke, heart failure, smoking, and alcohol use, along with admission clinical indicators such as blood pressure (systolic/diastolic) (15), Albumin (16), blood glucose, the NIHSS score, mRs score, GCS score, dysphagia (water-swallow tests grade II and below), disturbance of consciousness, presence of nasogastric tube and nasogastric tube resetion within 7 days. In addition, the location of occlusion (anterior circulation infarction or posterior circulation infarction), the leukoaraiosis degree (measured with the Fazekas scale:0–3, absent-mild–moderate–severe), EVT surgery time, post-treatment mTICI grade (grade 2b or above indicating vascular reperfusion), the length of mechanical ventilation time, days in the ICU, and postoperative complications (gastrointestinal bleeding, intracranial hemorrhage transformation, and brain herniation) were taken in our concern. Finally, it is believed that the Neutrophil-to-Lymphocyte Ratio (NLR), Systemic Immune Inflammatory Index (SII), Systemic Inflammatory Response Index (SIRI), and Platelet-to-Lymphocyte Ratio (PLR) are connected to the state of inflammation and infection risk (17–21), and they also have a correlation with post-stroke pneumonia (22). Hence, NLR, SII, SIRI and PLR within a day after the EVT mentioned above were incorporated as indicators.

### 2.4 Statistical analysis

To analyze clinical characteristics, continuous variables were represented as mean ± SD or median (IQR), depending on whether the data exhibited a normal distribution (determined by the Shapiro–Wilk test, with *p*>0.05). The dissimilarities among the two datasets were assessed utilizing either the non-parametric Mann–Whitney U test or the Student’s *t*-test. Frequencies and percentages (*n*, %) were used to present categorical variables, and the chi-square test or Fisher’s exact test was employed to estimate the differences between them. Statistical significance was determined when the two-sided *p*-value was less than 0.05.

To prevent overfitting in the analysis of this dataset with high dimensions, the Least Absolute Shrinkage and Selection Operator (LASSO) regression was utilized with tenfold cross-validation to identify possible predictive characteristics and determine the optimal lambda value (23). Subsequently, the variables identified via LASSO regression analysis were incorporated in the multivariable logistic regression analysis, employing a stepwise backward method, to further ascertain the factors that impact the incidence of VAP. Statistically significant clinical features were determined based on the multivariable analysis with *p*<0.05. The predictive factors were used to create a nomogram, where the outcomes were represented by Odds Ratio (OR), 95% Confidence Interval (95% CI), and *p*-values.

The performance of the model was evaluated by plotting the Receiver Operating Characteristic curve (ROC) and calculating the Area Under the ROC curve (AUROC), the value of which above 0.7 indicates a strong discriminative ability. Moreover, because of the small size of the sample in our research, in order to form reuse of data, the 10-fold cross-receiver operating characteristic (ROC) curves were performed for internal validation by using 9 of the 10 subsets for the stepwise backward regression and the last subset for validation to test the generalization error of the model. In addition, we used bootstrap resampling, with the number of resamples set to 1,000, and the calibration curve was plotted to depict the level of agreement between the observed and nomogram-estimated incidence of VAP. Meanwhile, the Hosmer-Lemeshow test for goodness-of-fit suggests that if the *p*-value is below 0.05, the model is poorly calibrated. Additionally, Decision curve analysis (DCA) was considered for the assessment of net benefit across different threshold probabilities in the dataset, which involved subtracting the proportion of false-positive cases from the proportion of true-positive cases and considering the negative consequences of both false-negative and false-positive interventions.

LASSO regression was performed using the R package named “glmnet 4.1.8”. DCA curves were created using the “rmda1.6” package, and ROC curves were generated using the “pROC 1.18.5” and “rms 6.7.1” package. SPSS version 25.0, R version 4.2.3, and Python version 3.11.4 were utilized for all analyses.

## 3 Results

### 3.1 Flowchart of study

**Figure 1:**
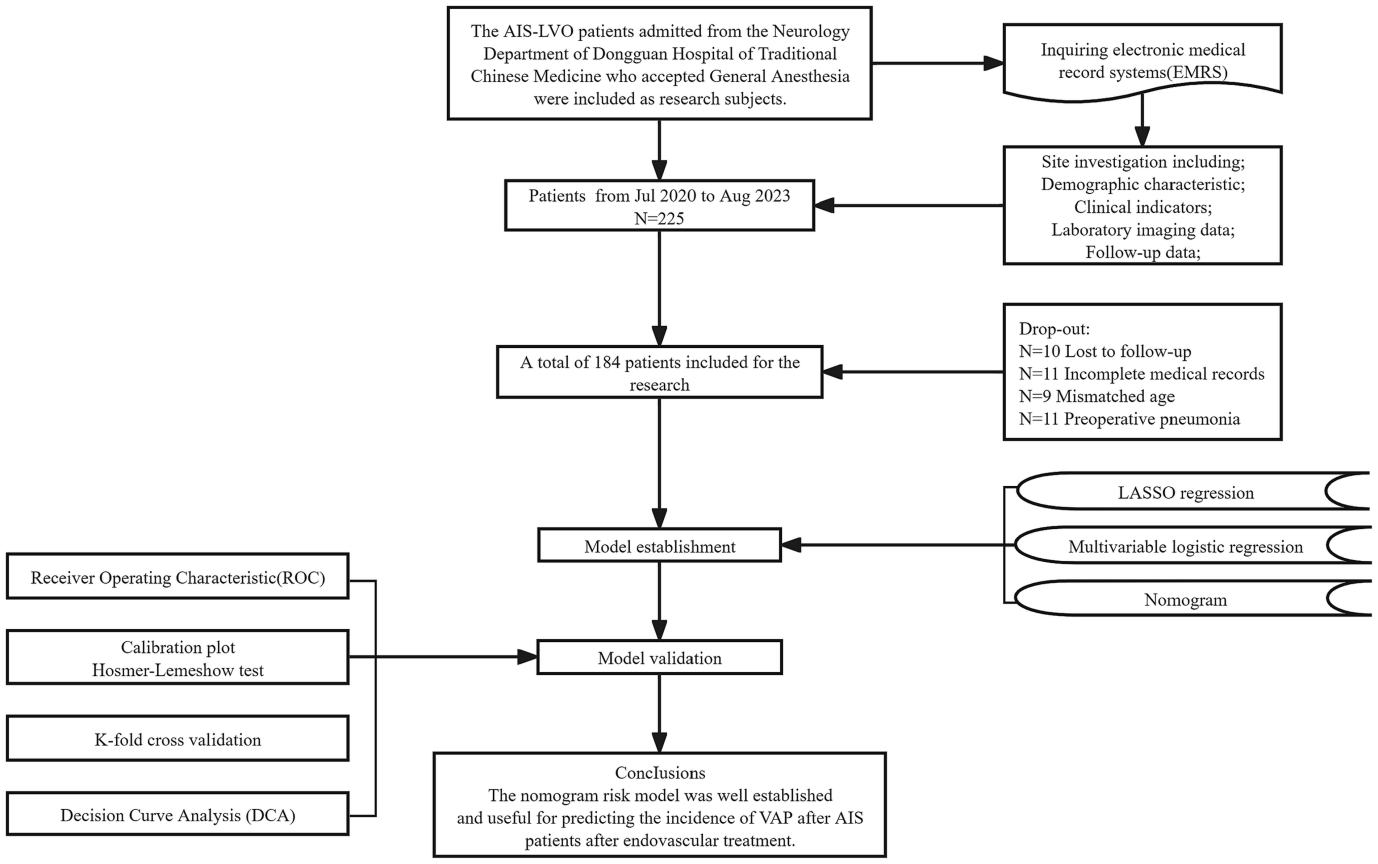
Flowchart.

The flowchart illustrating the enrolment process is shown in Figure 1. Of the 225 patients, 41 were eliminated not fulfilling the necessary criteria. Ultimately, a total of 184 individuals were enrolled in this research.

### 3.2 Baseline characteristics

**Figure 2:**
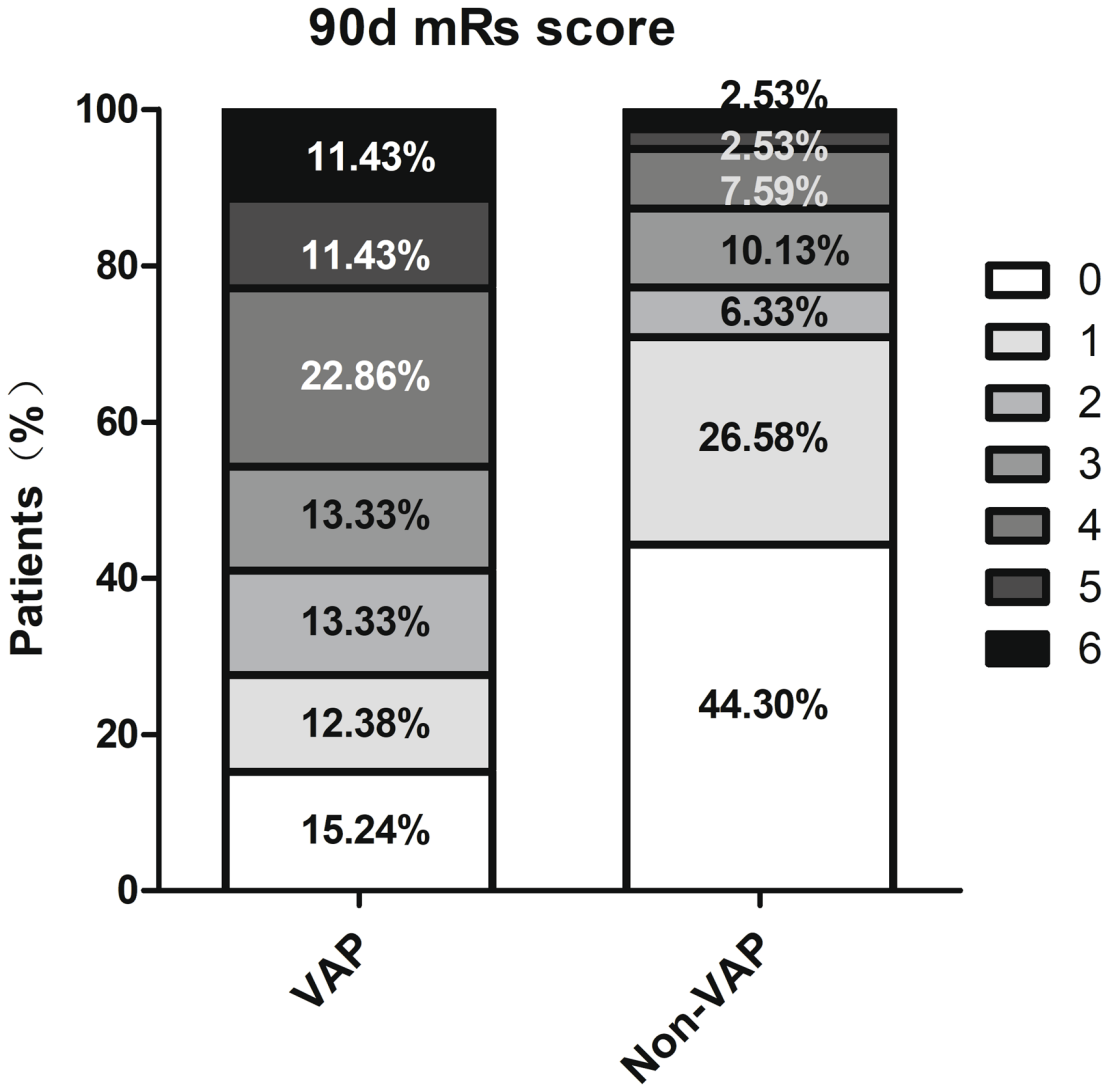
The distribution of the modified Rankin scale.

A total of 184 individuals (average age 61.85 ± 13.25 years, 73.37% male) were categorized into VAP and non-VAP groups depending on the occurrence of pneumonia within a week, with a VAP prevalence rate of 57.07%. Table 1 displayed the demographics and traits of both groups. Significant associations were found between the occurrence of VAP after EVT in AIS-LVO patients and factors such as age, diabetes, atrial fibrillation, disturbance of consciousness, dysphagia, Fazekas scale 1–3, admission NHISS score, mRs score (0–2), GCS score, ALB, postoperative SIRI, nasogastric intubation, duration of mechanical ventilation, and days in the ICU (*p*<0.05). Regarding the 90 days forecast, patients with VAP experienced a reduced occurrence of positive results (mRS 0–2) (40.95% vs. 77.22%, *p* < 0.001), along with higher mortality (11.43% vs. 2.53%, *p* = 0.024) (Figure 2).

**Table 1:** Demographics and characteristics of patients undergoing mechanical ventilation after EVT.

		Total (*n* = 184)	VAP group (*n* = 105, 57.07%)	Non-VAP group (*n* = 79, 42.93%)	*p-*value
Demographics					
Gender, male, *n* (%)		135 (73.37%)	73 (69.52%)	62 (78.48%)	0.174
Age, year, mean ± SD		61.85 ± 13.25	64.73 ± 12.80	58.01 ± 12.84	<0.001
**Medical history**					
Hypertension, *n* (%)		106 (57.61%)	63 (60.00%)	43 (54.43%)	0.449
Diabetes, *n* (%)		47 (25.54%)	33 (31.43%)	14 (17.72%)	0.035
Atrial fibrillation, *n* (%)		34 (18.48%)	26 (24.76%)	8 (10.13%)	0.011
Prior stroke, *n* (%)		36 (19.57%)	23 (21.91%)	13 (16.46%)	0.356
CAD, *n* (%)		28 (15.22%)	17 (16.19%)	11 (13.92%)	0.672
COPD, *n* (%)		2 (1.09%)	2 (1.91%)	0 (0.0%)	0.611
Heart failure, *n* (%)		5 (2.72%)	4 (3.81%)	1 (1.27%)	0.294
Smoking, *n* (%)		68 (36.96%)	35 (33.33%)	33 (41.77%)	0.240
Alcohol drinking, *n* (%)		36 (19.57%)	20 (19.05%)	16 (20.25%)	0.838
**Clinical features**					
Dysphagia, *n* (%)		69 (37.50%)	54 (51.43%)	15 (18.99%)	<0.001
Disturbance of consciousness, *n* (%)		62 (33.70%)	49 (46.67%)	13 (16.46%)	<0.001
Admission Systolic pressure, mmHg, mean ± SD		152.72 ± 23.36	155.16 ± 24.09	149.47 ± 21.93	0.103
Admission Diastolic pressure, mmHg, mean ± SD		89.61 ± 13.18	90.85 ± 13.35	87.98 ± 12.77	0.145
Admission NIHSS, median (IQR)		9 (4–15)	12 (6–16)	6 (3–11)	<0.001
Admission GCS, median (IQR)		15 (13–15)	15 (11–15)	15 (15–15)	<0.001
Admission mRs, 0 ~ 2 score, *n* (%)		46 (25.00%)	20 (19.05%)	26 (32.91%)	0.032
**Location of occlusion**					
Anterior circulation, *n* (%)		139 (75.54%)	80 (76.19%)	59 (74.68%)	0.814
Posterior circulation, *n* (%)		45 (24.46%)	25 (23.81%)	20 (25.32%)	
Fazekas scale, *n* (%)	0	100 (54.35%)	55 (52.38%)	45 (56.96%)	0.537
	1	47 (25.54%)	20 (19.05%)	27 (34.18%)	0.020
	2	28 (15.22%)	22 (20.95%)	6 (7.60%)	0.013
	3	9 (4.89%)	8 (7.62%)	1 (1.27%)	0.048
**Inspection results**					
Admission Glu ≥ 11.0 mmol/L, *n* (%)		20 (10.87%)	12 (11.43%)	8 (10.13%)	0.779
ALB, mmol/L, mean ± SD		38.35 ± 3.80	37.86 ± 3.99	39.00 ± 3.42	0.043
Admission WBC,10^9^/L, median (lQR)		8.99 (7.30–11.45)	8.83 (7.08–11.64)	9.07 (7.76–11.14)	0.735
Postoperative NLR, median (IQR)		6.92 (4.09–10.74)	6.70 (4.09–10.60)	7.45 (4.20–10.83)	0.915
Postoperative SII, median (IQR)		1473.27 (866.19–2518.45)	1436.66 (848.03–2398.54)	1637.93 (912.32–2624.76)	0.665
Postoperative PLR, median (IQR)		183.05 (131.49–278.63)	176.27 (132.41–284.71)	193.89 (131.49–267.29)	0.594
Postoperative SIRI, median (IQR)		1.80 (1.02–3.69)	1.98 (1.12–4.09)	1.54 (0.95–2.81)	0.018
**Procedure-related**					
Operation time, hour, median (IQR)		2.42 (2.00–2.83)	2.42 (2.00–2.92)	2.41 (2.00–2.83)	0.605
mTICI, *n* (%)	mTICI≥2b	178 (96.74%)	100 (95.24%)	78 (98.73%)	0.186
	mTICI<2b	6 (3.26%)	5 (4.76%)	1 (1.27%)	
**Postoperative complications**					
Intracranial hemorrhage, *n* (%)		25 (13.59%)	15 (14.29%)	10 (12.66%)	0.750
Gastrointestinal hemorrhage, *n* (%)		4 (2.17%)	4 (3.81%)	0 (0.0%)	0.217
Brain herniation, *n* (%)		2 (1.08%)	2 (1.91%)	0 (0.0%)	0.611
**Operations**					
Nasogastric intubation, *n* (%)		146 (79.35%)	92 (87.62%)	54 (68.35%)	0.001
Reset Nasogastric tube within 7 days, *n* (%)		7 (3.80%)	5 (4.76%)	2 (2.53%)	0.434
Duration of mechanical ventilation, hour, median (IQR)		18.34 (11.70–41.96)	23.25 (13.25–81.85)	13.48 (10.06–19.46)	<0.001
Days in ICU, median (IQR)		2.06 (1.51–3.76)	2.87 (1.81–5.88)	1.64 (1.33–2.45)	<0.001
**Prognosis**					
90d mRs, 0 ~ 2 score, *n* (%)		104 (56.52%)	43 (40.95%)	61 (77.22%)	<0.001
Mortality, *n* (%)		14 (7.61%)	12 (11.43%)	2 (2.53%)	0.024

IQR, interquartile range; CAD, coronary artery disease; COPD, chronic obstructive pulmonary disease; NIHSS, National Institute of Health stroke scale; GCS, Glasgow coma scale; mRs, modified Rankin scale; Glu, glucose; ALB, albumin; WBC, white blood cell; NLR, neutrophil-to-lymphocyte ratio = neutrophil count/lymphocyte count; SII, systemic immune inflammatory index = (neutrophil count × platelet count)/lymphocyte count; PLR, platelet-to-lymphocyte ratio = platelet count/lymphocyte count; SIRI, systemic inflammatory response index = (neutrophil count × monocyte count)/lymphocyte count; ICU, intensive care unit.

### 3.3 Nomogram construction

**Figure 3:**
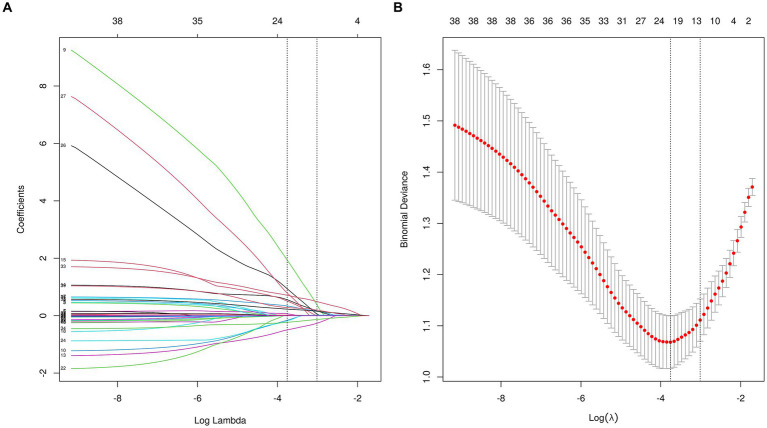
The LASSO regression was used to obtain optimal lambda parameter values for thirty-six features related to VAP-related risk factors. **(A)** Log(Lambda) value for the thirty-six characteristics in the LASSO regression. A plot of the coefficient profile was generated by plotting against the logarithmic sequence of lambda. The lambda value corresponding to the minimum mean square error is 0.023, while the lambda value for the standard error of the minimum distance is 0.049. **(B)** Curves for partial likelihood deviation (binomial deviation) and logarithmic (lambda) curves. Draw a vertical dashed line at the optimal value with the minimum standard and twelve variables had a regression coefficient that was not zero. LASSO, the least absolute shrinkage and selection operator.

A total of 36 variables underwent screening in the LASSO regression, employing 10-fold cross-validation to identify potential predictive characteristics. Consequently, 12 variables were selected to be significant predictors of VAP, with a minimum distance standard error λ of 0.049, and in order they were age, COPD, Fazekas scale 1, Fazekas scale 2, nasogastric intubation, postoperative gastrointestinal hemorrhage, dysphagia, GCS score, NIHSS score, days in the ICU, disturbance of consciousness, and admission diastolic blood pressure (Figure 3), which would subsequently be incorporated into multivariate logistic regression analysis.

**Figure 4:**
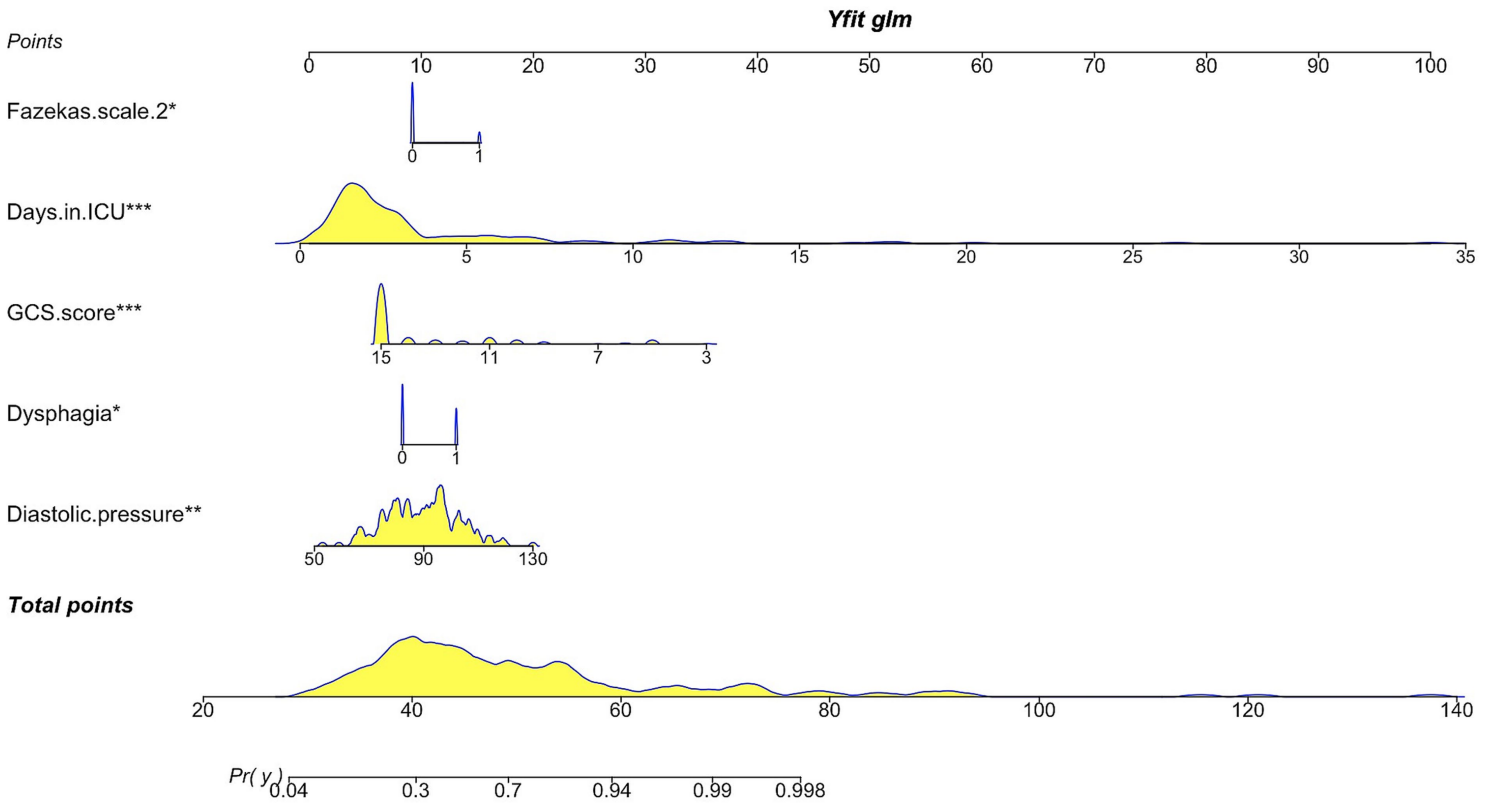
Nomogram illustrating the prediction of ventilator-associated pneumonia in postoperative mechanically ventilated patients after EVT. GCS score, Glasgow Coma Scale score.

Afterward, the multivariate logistic regression analysis with a stepwise backward elimination method revealed that dysphagia (Odds Ratio [OR], 2.51; 95% confidence interval [CI], 1.06–5.92; *p* = 0.036), diastolic blood pressure (OR, 1.05; 95%CI, 1.02–1.08; *p* = 0.003), GCS score (OR, 0.63; 95%CI, 0.49–0.81; *p*<0.001), Fazekas scale 2 (OR, 3.14; 95%CI, 1.00–9.85; *p* = 0.049) and days in the ICU (OR, 1.77; 95%CI, 1.33–2.35; *p*<0.001) remained independent predictors for the occurrence of VAP (Table 2). Therefore, the established predictive nomogram as shown in Figure 4, incorporates five variables above: Logit (P) = 0.559 + 0.920 × Dysphagia +0.047 × Diastolic pressure − 0.464 × GCS score + 1.145 × Fazekas scale 2 + 0.569 × Days in ICU. To determine the likelihood of developing postoperative VAP in patients, the total points were represented by the sum of the five variable points, and a perpendicular line was drawn from the total points axis to the outcome axis.

**Table 2:** Multivariate logistic regression analysis to ascertain the factors that impact the incidence of VAP.

Covariates	Multivariable		
	*β*	Odds ratio (95% CI)	*p*-value
(Intercept)	0.559	1.749 (0.029–146.745)	0.795
Dysphagia	0.920	2.509 (1.063–5.922)	0.036
Diastolic pressure	0.047	1.048 (1.016–1.081)	0.003
GCS score	−0.464	0.629 (0.491–0.806)	<0.001
Fazekas scale 2	1.145	3.144 (1.003–9.853)	0.049
Days in ICU	0.569	1.767 (1.328–2.351)	<0.001

### 3.4 Evaluation and clinical application of nomogram

**Figure 5:**
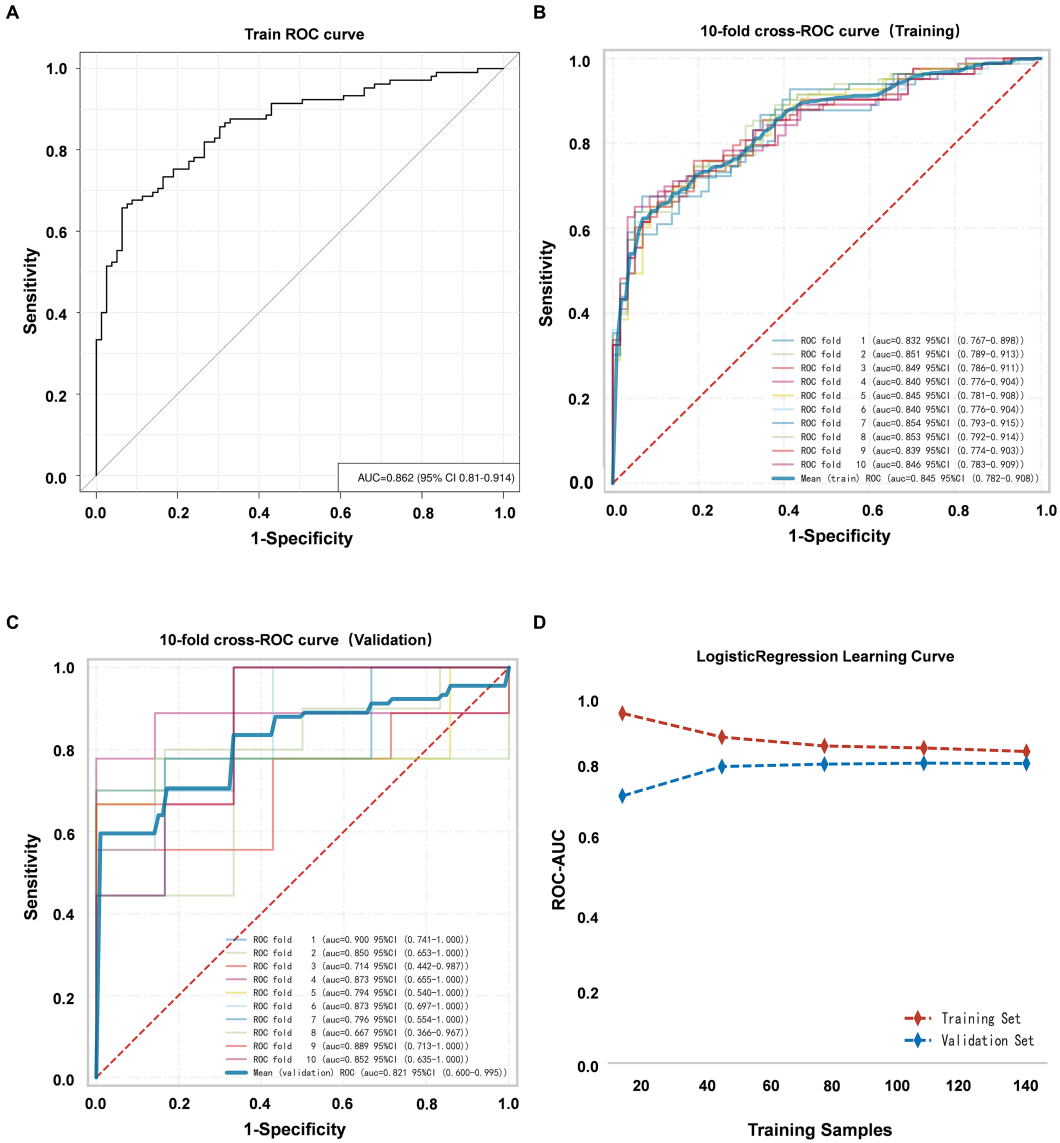
**(A)** ROC curve of the VAP prediction model. **(B)** The 10-fold cross-ROC curves for internal validation of the training set. **(C)** The 10-fold cross-ROC curves of the validation set. **(D)** The development of the AUC for the training set and validation set throughout the training of the logistic regression model.

The discriminative ability of the nomogram was evaluated by computing the AUC of the ROC curve, yielding a value of 0.862 (95% CI, 0.810–0.914) (Figure 5A). Furthermore, K-fold cross validation (*K* = 10) was utilized for internal validation of the model and all models have returned the same variables in k-fold cross validation. Additionally, the 10-fold cross-receiver operating characteristic (ROC) curves of the model under different cross-validation folds were performed (Figures 5B,C). The results showed a mean AUC of 0.821 (95% CI, 0.600–0.995) (Figure 5C), accuracy of 0.717 (95% CI, 0.679–0.754), sensitivity of 0.773 (95% CI, 0.688–0.859) and specificity of 0.869 (95% CI, 0.774–0.964) in the validation set. It is evident that the AUC of both the training and validation sets eventually stabilizes around 0.8, indicating strong predictive performance of the model during the training of the logistic regression model (Figure 5D). Moreover, the R-squared is 0.15 ± 0.41 and the mean square error is 0.186 ± 0.054 in the validation set.

**Figure 6:**
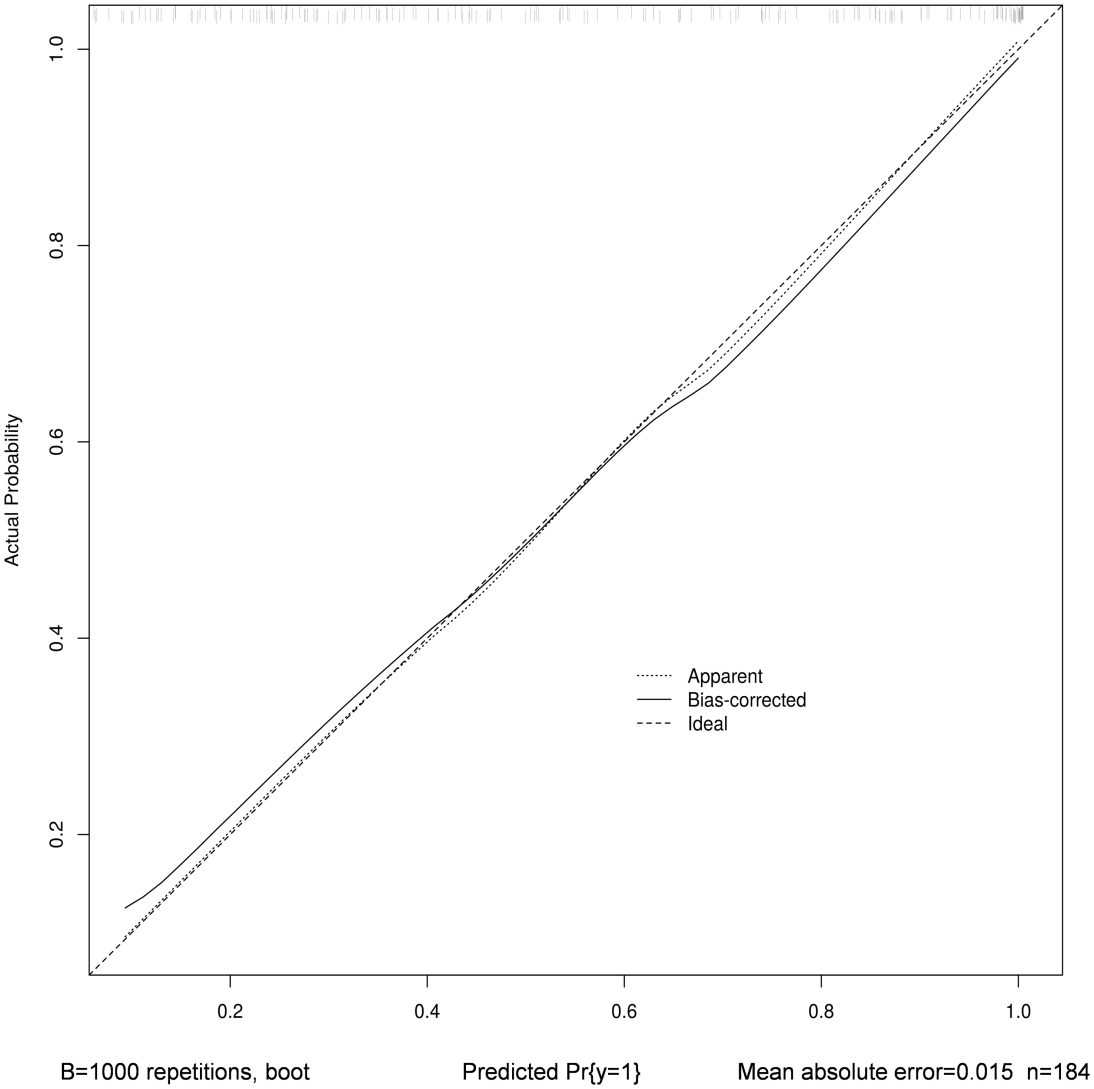
Calibration curve with bootstrap resampling 1,000 times of nomogram. The horizontal axis depicts the forecasted VAP vulnerability, while the vertical axis portrays the real diagnosis of VAP. The dashed line that runs diagonally represents a flawless forecast made by an optimal model. The performance of the column chart is represented by the solid line, and the prediction effect improves as it gets closer to the diagonal dashed line.

**Figure 7:**
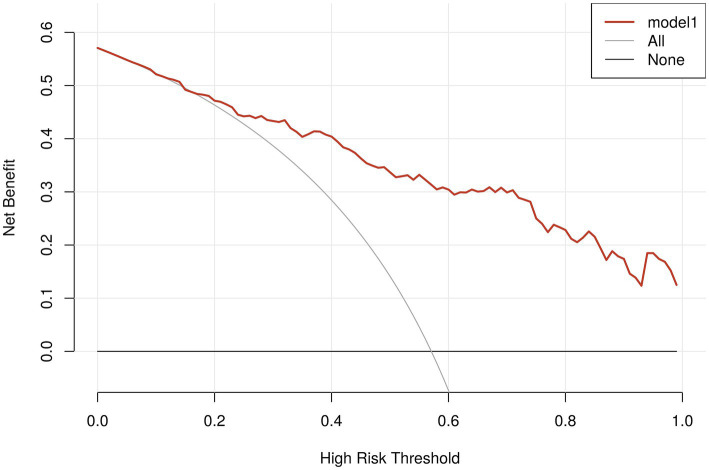
Analysis of decision curves for VAP-risk nomogram. The x-axis depicts the probability threshold, where the patient’s treatment should be initiated upon reaching a specific threshold. The y-axis represents the net clinical benefit for the patient receiving the treatment. The VAP clinical diagnostic model is represented by the red line in the figure, along with the two lines representing the two extreme cases. A sturdy, dense line depicts the assumption that no patients have VAP. The assumption that all patients have postoperative VAP is represented by a thin solid line.

The nomogram’s calibration was evaluated using the Hosmer-Lemeshow test (*p* = 0.723), which indicated a good fit. Additionally, the calibration curve after bootstrap resampling 1,000 times with mean absolate error = 0.015 showed a robust agreement (Figure 6). Ultimately, the Decision Curve Analysis (DCA) indicated that employing this nomogram for VAP prediction yields a higher overall advantage in comparison to both the “all intervention” and “no intervention” strategies, when the threshold probabilities surpass around 18%. It suggests the effectiveness of the nomogram, as demonstrated in Figure 7.

## 4 Discussion

In this research, the occurrence rate of ventilator-associated pneumonia (VAP) among large vessel occlusion stroke patients after endovascular treatment (EVT) was 57.20%, surpassing the prevalence reported earlier. Patients diagnosed with acute ischemic stroke with large vessel occlusive (AIS-LVO) experience a critical state due to the need for invasive procedures, impaired awareness, difficulty swallowing, and admission to the intensive care unit (ICU), all of which contribute to a heightened risk of pneumonia. Tracheal intubation exposes the original relatively sterile lower respiratory tract directly to the outside world. The challenge of oral hygiene enhances the proliferation of colonizing bacteria in the oropharynx, leading to the flow of oral secretions that contain significant quantities of colonizing bacteria into the lower respiratory tract (24). Simultaneously, the existence of tracheal intubation hinders the patient’s ability to cough efficiently and diminishes their capacity to eliminate phlegm. Furthermore, the tracheal tube is susceptible to the development of biofilms on both its inner and outer surfaces, which can detach due to various factors like sputum suctioning (25). The blockage of the narrow air passages can increase the chances of developing pneumonia Patients diagnosed with AIS-LVO frequently experience reduced awareness, difficulty swallowing, and extended periods of immobility, which heightens their susceptibility to unintentional inhalation. Consequently, the coexistence of stroke and mechanical ventilation frequently amplifies the likelihood of pneumonia development in the individual. Besides administering active anti-infective therapy following the onset of VAP, it is crucial from a clinical standpoint to decrease the occurrence of VAP through enhancing airway safeguarding and promptly identifying and intervening with targeted measures. Similar to previous research (3, 5, 26), our findings indicate that patients who experienced pneumonia following EVT had a worse functional prognosis and a higher mortality rate.

The univariate analysis in this study found a significant association between VAP after EVT and factors such as age, diabetes, atrial fibrillation, impaired consciousness, dysphagia, Fazekas scale 1–3, NIHSS score, GCS score, mRs score, postoperative SIRI, ALB, indwelling gastrostomy tubes, duration of mechanical ventilation, and length of ICU stay, which align with previous studies (20, 27–29). It is important to mention that the significance of nutritional elements in infectious incidents among patients who are hospitalized has progressively gained extensive recognition in recent times. Nevertheless, the findings of our research revealed that individuals diagnosed with pneumonia exhibited reduced levels of albumin. The patient’s overall immune function may be reflected by the albumin status upon admission, and the occurrence of immune suppression and the promotion of lung infections in patients may be caused by hypo-proteinaemia (16). Moreover, this research incorporated four parameters (NLR, SII, PLR, and SIRI) to examine the correlation with VAP, among which only the postoperative SIRI exhibited a significant association with the onset of VAP. The marker SIRI has received attention because it can indicate the overall inflammatory condition and immune reaction as well as exhibit a strong capacity to predict pneumonia in individuals suffering from acute ischemic stroke, as shown in previous studies (20, 22, 30).

ICU stay duration, Glasgow Coma Scale (GCS) score, dysphagia, Fazekas scale 2 and admission diastolic blood pressure were identified as autonomous factors affecting the development of VAP in the multifactorial analysis results. After the EVT procedure, the majority of patients will be moved to intensive care units (ICUs) or neurological intensive care units (NICUs) for additional monitoring. It is evident that the development of pneumonia is closely associated with the hospital environment because ICU wards are typically enclosed areas with inadequate airflow, and the exhaled gases and secretions of ICU patients contain numerous pathogens, leading to air pollution. The risk of infection rises as the duration of hospital stay increases (31). Therefore we need to make efforts to shorten extubation and ICU hospitalization time while urgently improving reperfusion. In clinical practice, the Glasgow Coma Score (GCS score) is frequently employed to evaluate the level of coma and the seriousness of craniocerebral injury in patients. Lower scores suggest greater impairment of consciousness and a worse prognosis. This study’s findings align with previous research indicating that it is also associated with early infections following a stroke (32).

Patients diagnosed with AIS-LVO often present with impaired consciousness, dysphagia, and extended periods of immobility, especially in patients with posterior circulation infarction, rendering them highly prone to aspiration (33–35). A primary risk factor for pneumonia is post-stroke dysphagia (PSD), which causes aspiration of food, fluids, and oral secretions into the lungs (36). Studies have shown that approximately half of all stroke patients experience aspiration, patients with dysphagia who have had a stroke are at an increased risk of pneumonia, which is eight times greater compared to patients without dysphagia (37). Furthermore, the mortality rate of stroke patients with dysphagia was 3 times higher than patients without dysphagia (38). Many hospitals throughout the world use dysphagia screening protocols to identify patients at risk of aspiration and to guide subsequent diagnostic and therapeutic procedures. Recent studies reported higher stroke-associated pneumonia in stroke patients who failed high-sensitive screening for dysphagia compared to those who passed the screening (39). However, higher stroke-associated pneumonia in stroke patients who passed low-sensitive screening for dysphagia compared to those who passed high-sensitive ones who can detect also silent aspiration (40). Both the gold standard the Videofluoroscopic Swallowing Study (VFSS) and the Flexible Endoscopic Evaluation of Swallowing (FEES) have practical limits to be routinely performed in the acute phase. In our research, water-swallow tests (WSTs) are used to screen patients with dysphagia, usually by having the patient drink a predetermined volume of water (e.g., 50 or 90 mL). The test was considered positive whenever clinical signs of aspiration (cough, voice change, wheezing) were present during or after screening. However, we neglected the silent aspiration, which is also a limitation of our study, because the detection of silent aspiration which is defined as aspiration without cough or other signs of distress, relies on the use of techniques other than bedside clinical assessment (40).

For AIS-LVO patients with dysphagia, difficult extubation from mechanically ventilated trachea, insufficient oral intake, or impaired consciousness, authors perform a thorough assessment of patients with dysphagia to select the appropriate method of feeding and preferentially recommend nutritional support by nasogastric tube (NGT) (41). For patients who refuse nasogastric tube placement, authors would help patients reduce aspiration by increasing the concentration of liquid and food by adding solidified powder, postural techniques (such as adopting a chin tuck position), and swallow strategies (such as a supraglottic swallow). NGT is an effective way to provide nutritional supply for patients with dysphagia, but there are many disadvantages of NGT tube feeding: This can cause bacterial translocation in the pharynx or lead to muscle atrophy of the tongue, palate, pharynx, and esophagus, hindering the recovery of swallowing function (42). Of utmost importance, it could result in incomplete closure of the cardia located at the base of the stomach and relaxation of the lower esophagus, increasing the patient’s vulnerability to food regurgitation and aspiration, ultimately resulting in lung infections (43). These results indicated that an indwelling gastric tube was significantly associated with the occurrence of VAP in stroke patients after EVT, which is consistent with prior research. In addition, because displacement and repositioning of the nasogastric tube is an associated risk factor for the development of pneumonia, we included data on the replacement of the nasogastric tube within 7 days to explore the correlation with the occurrence of VAP, but no statistically significant difference was found in our research.

Similarly to ICU stay duration, GCSscore and dysphagia, the leukoaraiosis degree (measured with the Fazekas scale) is a common finding among large vessel occlusion stroke patients (44), and has been described as a significant predictor of poor outcome after acute acute ischemic stroke (45, 46). Therefore we included the leukoaraiosis degree as a reference factor for modeling. The cerebral leukoaraiosis was classified using the Fazekas score, with grades 0–1 indicating absent-mild and grades 2–3 indicating moderate–severe. Our univariate analyses confirmed that Fazekas scale 1–3 was statistically significantly different between the VAP group and the non-VAP group (*p*<0.05), and that there was a higher Fazekas score for patients with VAP. Previously reported excess leukoaraiosis in women with TIA/stroke is likely to be confounded by age (47). In our multifactorial analyses, patients with Fazekas grade 2 showed significant differences after excluding confounding factors such as age, suggesting that the leukoencephalopathy is an independent influence on the development of VAP after endovascular treatment of acute large vessel occlusion cerebral infarction. Therefore we included the variable of which Fazekas grade 2 in our model. Among stroke patients, LA has been associated with the dimension of the ischemic core volume in the hyperacute phase, the dimension of the final cortex infarct volume, and the growth of the acute ischemic lesion (48–50). There are several mechanisms involved (51–53), including endothelial dysfunction, micro-vessel density reduction, decreased collateral flow, and reduction in functional reserve.

It is important to mention that our findings have identified that admission diastolic blood pressure could potentially serve as a VAP-risk predictive factor. Some studies have suggested that elevated blood pressure might be linked to a higher risk of developing pneumonia (15, 54, 55). Harms et al. study (56) proposed that an increase in systolic blood pressure exceeding 200 mmHg within 24 h of hospital admission could be expressed as a sign of this overactivation. Nevertheless, there is little reported evidence suggesting that diastolic blood pressure has an individual impact on the occurrence of pneumonia, unlike systolic blood pressure. Only one study using Mendelian randomization mentioned a notable rise in pneumonia risk for each 5 mmHg increase in systolic and diastolic blood pressure (HR 1.08, *p* < 0.001 vs. HR 1.11, *p* = 0.005) (15). High blood pressure could potentially contribute to the onset of infection through various mechanisms, and it may also worsen pulmonary stasis or exacerbate the disruption of adaptive immune responses in patients after surgery. Molecular pathway analyses suggest that elevated blood pressure may induce inflammatory pathways (54, 57) and lead to endothelial dysfunction promoting pneumonia (58). Additionally, an increase in blood pressure can lead to the development of pneumonia due to its negative impact on lung function. Jankowich et al. (59) discovered a notable inverse relationship (−0.37) between forced expiratory volume in 1 s (FEV1) and average pulse pressure. Admission diastolic blood pressure, as an easy-to-monitor hemodynamic index, has the advantages of clinical convenience of dynamic observation and intervention, through early identification, early active intervention may be able to make these critically ill patients get better prognosis. However, according to our observation, researchers pay less attention to diastolic blood pressure in the clinic and scientific research. Further investigation is warranted to explore the possible ways in which diastolic blood pressure contributes to the onset of pneumonia.

In this study, both internal validation and the Hosmer-Lemeshow test demonstrated that the nomogram greatly reflected the real-life situation. Additionally, the results of the ROC analysis indicated that the prediction model had a good predictive value for the occurrence of VAP in EVT patients, with an AUC of 0.862. The nomogram was developed to assist neurologists in identifying patients with VAP after endovascular treatment. Clinicians can mitigate the risk of VAP by directing identifications and interventions toward individuals with pertinent attributes, thereby enhancing patients’ prognosis.

## 5 Conclusion

In acute ischemic stroke patients after endovascular treatment, the nomogram, consisting of the length of ICU stay, GCS score, dysphagia, Fazekas scale 2 and diastolic blood pressure upon admission, has the potential to forecast the development of VAP. This Nomogram graphical prediction model allows for personalized and graphical predictions of VAP development in AIS-LVO patients undergoing EVT, which means early identification enables timely and effective treatment, improving outcomes of patients.

## 6 Limitations

This study has multiple constraints. (1) Initially, the study was carried out retrospectively at a solitary facility, implying the possibility of unaccounted variables and potential bias in participant selection. (2) Additionally, the sample size used in the study was limited. Hence, it is necessary to conduct larger trials involving multiple centers or conduct external validation with a more extensive sample size to validate the dependability of this model. (3) In this research, the dysphagia screening conducted at the bedside identified the majority of evident dysphagia cases. However, we did not utilize more specialized criteria such as the Videofluoroscopic Swallowing Study (VFSS) and Flexible Endoscopic Evaluation of Swallowing (FEES), consequently resulting in the omission of some patients with silent aspiration. Although overt dysphagia is a common feature in patients with stroke combined with pneumonia, several studies give prevalence estimates for silent aspiration in subacute dysphagia stroke patients of 15–39% (60–62), suggesting that a large proportion of pneumonia patients may be due to silent aspiration. This datum dramatically affects the PSD patients both dysphagia detection and its prognosis (39, 40). Silent aspiration might associated with reduced pharyngeal sensation and an impaired, low cough reflex (40). Aviv et al. (63) found that 11/18 stroke patients without clinical dysphagia had impaired pharyngeal sensation during an air-pulse test on the mucosa innervated by the superior laryngeal nerve. In addition, it is also possible that prolonged tracheal intubation impairs vocal cord movement (64), and airway desensitisation leads to silent malabsorption. (4) Due to the retrospective study, no data were gathered on HLA-DR and IL-6, which have notable correlations with stroke-associated pneumonia (SAP).

## Data availability statement

The raw data supporting the conclusions of this article will be made available by the authors, without undue reservation.

## Ethics statement

The studies involving human participants were reviewed and approved by the Ethics Committee of Dongguan Hospital, Guangzhou University of Chinese Medicine (PJ [2023] No. 63). Written informed consent from the patients/participants or patients/participants’ legal guardian/next of kin was not required to participate in this study in accordance with the national legislation and the institutional requirements.

## Author contributions

H-sZ: Software, Validation, Visualization, Writing – original draft, Writing – review & editing, Conceptualization, Data curation, Formal analysis, Methodology. W-fL: Data curation, Formal analysis, Investigation, Writing – original draft, Writing – review & editing. J-lZ: Data curation, Formal analysis, Visualization, Writing – original draft. X-hH: Conceptualization, Data curation, Formal analysis, Investigation, Writing – original draft. P-jZ: Data curation, Formal analysis, Investigation, Writing – original draft. K-qY: Conceptualization, Data curation, Formal analysis, Investigation, Writing – original draft. G-sL: Data curation, Formal analysis, Investigation, Writing – original draft. BL: Data curation, Formal analysis, Investigation, Project administration, Writing – original draft. H-qD: Data curation, Formal analysis, Investigation, Project administration, Writing – original draft. Z-cL: Data curation, Formal analysis, Investigation, Writing – original draft. J-sZ: Data curation, Formal analysis, Project administration, Writing – original draft. ZZ: Investigation, Methodology, Writing – review & editing. J-yC: Project administration, Supervision, Writing – review & editing. Q-xH: Data curation, Funding acquisition, Supervision, Validation, Writing – review & editing. W-mN: Conceptualization, Data curation, Funding acquisition, Project administration, Resources, Supervision, Writing – review & editing.
